# The secreted proteases *aur, scpA, sspA and sspB* suppress the virulence of *Staphylococcus aureus* USA300 by shaping the extracellular proteome

**DOI:** 10.1080/21505594.2025.2514790

**Published:** 2025-06-11

**Authors:** Xiaofang Li, Sandra Maaß, Borja Ferrero-Bordera, Zhichao Zhang, Min Wang, Eric Sietsema, Lei Liu, Madeleine Divinagracia, Jan Maarten van Dijl, Girbe Buist

**Affiliations:** a Department of Medical Microbiology and Infection Prevention, University of Groningen, University Medical Center Groningen, Groningen, the Netherlands; b Department of Microbial Proteomics, University of Greifswald, Centre of Functional Genomics of Microbes, Institute of Microbiology, Greifswald, Germany; c Institute of Medical Psychology, LMU Munich, Munich, Germany

**Keywords:** *Staphylococcus aureus*, virulence, proteases, peptidoglycan hydrolase, cell lysis

## Abstract

Surface-located and secreted virulence factors of the Gram-positive bacterial pathogen *Staphylococcus aureus* are key drivers for infection of the human host. Proteolytic enzymes may contribute to virulence by breaking primary barriers and host immune defenses. Therefore, the objective of our study was to chart the contributions of different proteases to *S. aureus* virulence, and to assess their roles in shaping the staphylococcal surface proteome (the “surfacome”) and extracellular proteome (the “secretome”). To this end, we applied 12 protease mutants of the *S. aureus* USA300 lineage. Four mutants lacking the metalloprotease aureolysin (Aur), the cysteine proteases staphopain A (ScpA) and SspB, or the serine protease SspA displayed enhanced cytotoxicity toward human lung epithelial cells, showing that they serve to suppress virulence. Profiling of the surfacomes and secretomes of the four mutants allowed correlation of their increased cytotoxicity to altered virulence factor profiles. Furthermore, enhanced levels of virulence factors were detected in the mutants’ surfacomes, which was shown to be relevant as all four mutants displayed enhanced lung epithelial cell invasion. Enhanced levels of cytoplasmic and membrane proteins in the mutant’s surfacomes showed that Aur, ScpA, SspA and SspB set limits to autolysis by reducing the levels of peptidoglycan hydrolases. We conclude that Aur, ScpA, SspA and SspB have key roles in shaping the surfacome and secretome of *S. aureus*, thereby controlling the virulence of this major pathogen. This implies that novel antimicrobial agents or vaccines should not target these proteases.

## Introduction

*Staphylococcus aureus* is a versatile Gram-positive bacterial pathogen that is asymptomatically carried by about 30% of the human population [1,2]. The main *S. aureus* reservoirs in humans are the nose, throat, skin, gut and perineum [3,4]. Infections caused by *S. aureus* can range from minor skin infections to severe bacteremia, sepsis and pneumonia. To colonize and invade the human body, *S. aureus* relies on a wide range of virulence factors, which play important roles in adhesion to the skin, mucosa and tissues of the host, passage of the epithelial and endothelial barriers, as well as evasion from the immune system [5,6]. Generally, these virulence factors can be categorized based on their cellular or extracellular localization. Proteins located on the pathogen’s surface are collectively referred to as the “surfacome” [7]. With respect to virulence, surfacome proteins of *S. aureus* play major roles in the bacterial adherence to extracellular matrices of host cells and other host ligands, as well as host invasion and immune evasion [8]. In contrast, secreted proteins of *S. aureus*, collectively referred to as the secretome, destroy the host’s primary barriers through the disruption of cell membranes, and they help to evade and kill immune cells [6].

Proteolytic enzymes represent a specific group of secreted virulence factors that can degrade proteins of the host during the different stages of invasive disease [9–11]. *S. aureus* secretes a diverse cocktail of proteases, including staphylococcal serine proteases and serine protease-like proteins (e.g. SspA, HtrA, SplA, SplB, SplC, SplD, SplE and SplF), cysteine proteases (e.g. staphopain A [ScpA] and SspB), and the metalloprotease aureolysin (Aur). In addition to these extracellular proteases, the intracellular caseinolytic protease P (ClpP) and the carboxyl-terminal protease (CtpA) are important proteases that have been implicated in cellular protein quality control, stress responses, regulation of virulence, and antibiotic resistance [12]. Among the extracellular proteases, only the Spl enzymes coded by the *splA-F* genes do not require proteolytic activation [13]. In contrast, the Aur, ScpA, SspA and SspB proteases are produced as so-called zymogens or pro-enzymes that require proteolytic activation [14,15]. The Aur and ScpA proteases help the bacteria to evade the host’s immune defenses and to invade host cells. For instance, it was reported that ScpA is involved in degrading fibrinogen and collagen [16]. In addition, secreted proteases also play roles in biofilm formation through degradation of the peptidoglycan hydrolase (PGH) Atl [17,18].

Interestingly, it was recently proposed that extracellular proteases of *S. aureus* can also selectively degrade the bacterium’s own virulence factors, thereby influencing the progression of infection [19]. In particular, it was shown that a strain lacking all extracellular proteases presented a hyper-virulent phenotype in a murine sepsis model, which was attributed to the accumulation of virulence factors. Further research showed that the leukocidin LukA and the secreted SAUSA300_0964 protein were main contributors to the increased virulence of a strain that lacked the Aur and ScpA proteases. This implies that Aur and ScpA play important roles in the turnover of secreted *S. aureus* virulence factors [19]. Conversely, other studies reported that increased production of extracellular proteases in the highly virulent *S. aureus* USA300 strain, which was triggered by a *sarA* mutation, led to a significant decrease in the number of identified extracellular proteins, including some important virulence factors, such as protein A (SpA), α-toxin and thermonuclease [20,21]. Furthermore, elevated levels of Aur and ScpA were associated with reduced virulence in a murine osteomyelitis infection model. In a previous study on *S. aureus* isolates with the *spa*-type t437, we associated the degradation of secreted virulence factors, such as the immunodominant staphylococcal antigen A (IsaA) and the staphylococcal complement inhibitor (SCIN) with increased levels of the Aur, ScpA and SsaA1 proteases [22]. Interestingly, we observed that also intracellular proteases can contribute to the degradation of secreted IsaA [23].

IIn addition to virulence, surfacome proteins play vital roles in maintaining the integrity of bacterial cells, the uptake of nutrients, bacterial growth and bacterial cell division [24]. In addition, they contribute to the communication between bacteria and their environment. The association of proteins with the bacterial cell surface is achieved via different mechanisms, including anchoring to the membrane by a transmembrane domain or a lipid modification, covalent attachment to the cell wall peptidoglycan by specific transpeptidases known as sortases, or non-covalent attachment through cell wall-binding domains or ionic interactions [8]. Although these different mechanisms effectively retain proteins at the bacterial cell surface, such proteins are also released into the external environment of the bacteria as exemplified by the non-covalently cell wall attached protein IsaA [25,26]. This shedding of proteins from the bacterial cell surface into the extracellular environment may be caused by cell lysis, but it may also be a consequence of regular cell wall remodeling during bacterial growth and division, or to cleavage by proteases that are secreted by the bacteria [24].

ISince proteins on the bacterial cell surface have key roles in virulence and are also potential targets for novel antibiotics and vaccines [27,28], it is important to understand which factors determine their surface exposure, integrity and possible release into the extracellular environment. This was the incentive for our present study, which was specifically aimed at (i) comparing the contributions of different proteases to the virulence of *S. aureus*, and (ii) assessing the roles of these proteases in shaping the staphylococcal surfacome and secretome. To this end, we applied a collection of protease mutants of the notorious community-associated *S. aureus* USA300 lineage. *S. aureus* USA300 was selected for this study based on its high virulence, methicillin resistance and well-characterized genetic background [29,30], which makes it an ideal model for studying virulence mechanisms and mutation-associated variations at the proteome level [20,31–33].

## Materials and methods

### Bacterial strains and culturing

**Figure 1.**
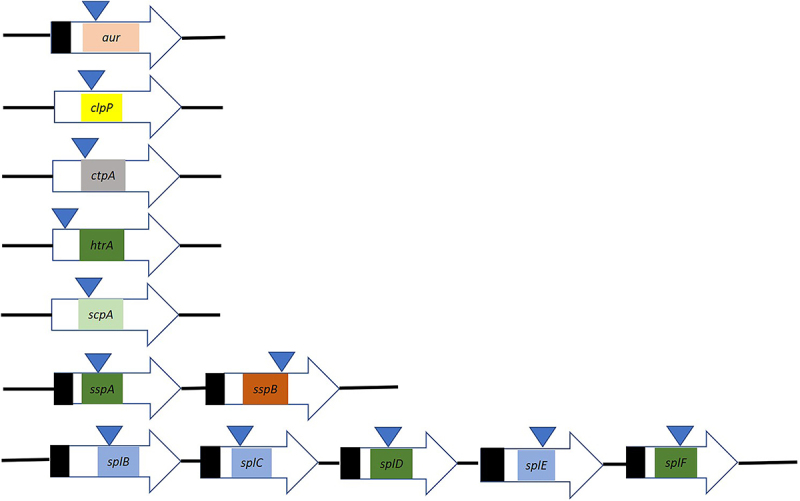
Genetic organization of protease genes of *S. aureus* USA300 and the respective transposon insertions. The genes are represented by horizontal arrows and transposon insertions are marked by vertical arrows. The black boxes indicate the signal peptides of the respective proteases. Color-coded boxes mark the functional proteolytic domains as defined by Pfam and indicated in UniProt: (pink, PF02868: thermolysin metallopeptidase, alpha-helical domain; yellow, PF00574: clp protease; light gray, PF01471: putative peptidoglycan binding domain; green, PF13365: trypsin-like peptidase domain; light green, PF02616: segregation and condensation protein ScpA; orange, PF14731: staphopain proregion; light blue, PF00089: trypsin).

The *S. aureus* USA300 wild-type (WT) and protease mutant strains from the Nebraska transposon mutant library used in this study are listed in Table 1. The transposon insertion sites in individual protease genes are schematically represented in Figure 1. As shown by previous whole-genome sequencing the respective transposon insertions were stably maintained under the applied culture conditions [38,39]. For routine culturing and proteomics analyses, the bacteria were grown in Tryptic Soy Broth (TSB, Oxoid Limited, Hampshire, UK) at 37°C under vigorous shaking (250 rpm). For the cytotoxicity and epithelial cell infection experiments, the bacteria were cultured in Roswell Park Memorial Institute 1640 medium (RPMI; Gibco, New York) supplemented with 2 mm L-glutamine (Thermo Fisher Scientific, Waltham, USA). RPMI medium was used, because it was previously shown through genome-wide transcript profiling that the transcriptome of *S. aureus* grown on RPMI closely resembled the transcriptome of *S. aureus* grown in human plasma [40]. This implies that RPMI closely mimics the (iron-restricted) conditions that *S. aureus* encounters during invasive infections.

**Table 1.** *1052” ia_version=“0”>S. aureus* strains used in this study.

Protease mutation	Strain designation*	Genotype^#^	Reference
*aur*	NE163	USA300 JE2, transposon in SAUSA300_2572, Em^R^	[34]
*clpP*	NE912	USA300 JE2, transposon in SAUSA300_0752, Em^R^	[34]
*ctpA*	NE847	USA300 JE2, transposon in SAUSA300_1313, Em^R^	[34]
*htrA*	NE881	USA300 JE2, transposon in SAUSA300_0923, Em^R^	[34]
*scpA*	NE1740	USA300 JE2, transposon in SAUSA300_1445, Em^R^	[34]
*sspA*	NE1506	USA300 JE2, transposon in SAUSA300_0951, Em^R^	[34]
*sspB*	NE934	USA300 JE2, transposon in SAUSA300_0950, Em^R^	[34]
*splB*	NE1207	USA300 JE2, transposon in SAUSA300_1757, Em^R^	[34]
*splC*	NE1098	USA300 JE2, transposon in SAUSA300_1756, Em^R^	[34]
*splD*	NE1577	USA300 JE2, transposon in SAUSA300_1755, Em^R^	[34]
*splE*	NE1844	USA300 JE2, transposon in SAUSA300_1754, Em^R^	[34]
*splF*	NE1764	USA300 JE2, transposon in SAUSA300_1753, Em^R^	[34]
-	USA300 LAC	USA300 wild-type	[35]
-	AH1263	USA300 LAC wild-type, Em^S^	[36]
*aur*, *scpA*, *sspA*, *sspB*	AH1919	USA300 ∆*aur*, ∆*scpA*, ∆*sspAB*	[37]

*NE numbers relate to strain numbering in the Nebraska transposon mutant library [34]; ^#^UniProt accession codes for the transposon-disrupted protease genes of *S. aureus* USA300 are presented; Em^R/S^, erythromycin resistant/sensitive.

### Cell line and culture

The human lung epithelial cell line 16HBE14o- (Sigma-Aldrich, Germany [41]) was used for infection experiments. The eukaryotic minimal essential medium (eMEM; Biochrom AG, Berlin, Germany), supplemented with 10% (v/v) fetal calf serum (Biochrom AG), 1% (v/v) sodium pyruvate (Sigma-Aldrich, Germany) and 1% (v/v) non-essential amino acids (PAN-Biotech GmbH, Germany), was used for cell culture (37°C with 5% CO_2_). The medium was changed every two days, and the cells were split every three days with 0.25% trypsin-EDTA (Gibco, Grand Island, USA). To avoid the loss of primary cell characteristics, the presently used cells underwent less than 10 passages.

### LDH cytotoxicity tests

To assess the cytotoxicity of the different bacterial strains toward 16HBE14o- cells, the lactate dehydrogenase (LDH) cytotoxicity assay kit of Thermo Fisher Scientific was used according to the manufacturer’s instructions and as previously described [38]. Briefly, the optimum cell number (1 × 10^4^) was determined before each experiment. For the main experiment, triplicate wells of a 96-well tissue culture plate were seeded with 1 × 10^4^ cells that had been resuspended in 100 μl eMEM. The cells were then incubated overnight at 37°C and 5% CO_2._ On the day of cytotoxicity testing, bacteria cultured overnight in TSB were diluted with RPMI medium to an optical density at 600 nm (OD_600_) of 0.06. Culturing was continued in a shaking water bath at 150 rpm until the bacteria reached the exponential phase (OD_600_ ~ 0.5). To synchronize the bacterial growth, the bacterial cultures were diluted once more to an OD_600_ of 0.06 in RPMI medium and culturing was continued overnight. Before the cytotoxicity assay was initiated, the number of 16HBE14o- cells in one well was counted using a Luna-II Automated Cell Counter (Westburg, Leusden, the Netherlands). Subsequently, 10 μl of each cultured bacterial strain were added to the triplicate wells with the cultured cells to achieve a multiplicity of infection (MOI) of 25. Two control groups were included in the analyses. Firstly, to assess spontaneous LDH release, one set of triplicate wells with cells was supplemented with 10 µL of sterile water. Secondly, to measure maximal LDH activity, another set of triplicate wells with cells was supplemented with 10 µL of 10× lysis buffer (1× at final concentration). The plate was then incubated at 37°C with 5% CO_2_ for 1 h. Thereafter, the samples were transferred to a clean flat-bottom 96-well plate, 50 µL of reaction mixture was added to each sample, and the plate was incubated in the dark for 30 min at room temperature (RT). Lastly, each well was supplemented with 50 µL of stop solution and the absorbance at 490 nm and 680 nm was determined. LDH activity was determined by subtracting the 680 nm absorbance value (background) from the 490 nm absorbance value. The percentage of cytotoxicity was calculated by using the following formula: % cytotoxicity = [(Bacteria-treated LDH activity-spontaneous LDH activity)/(maximum LDH activity- spontaneous LDH activity)] × 100. The LDH cytotoxicity assays were performed as independent biological triplicates.

### LDS-PAGE and Western blotting

*S*. *aureus* bacteria in the exponential growth phase were obtained by inoculation of fresh TSB medium with bacteria from an overnight culture in TSB and subsequent cultivation for ~3 h at 37°C under vigorous shaking (250 rpm) until an OD_600_ of 0.5 was reached. Bacteria in the stationary growth phase were obtained from the overnight cultures in TSB. To facilitate processing of bacterial samples for protein extraction and subsequent analyses, the overnight cultures were adjusted to an OD_600_ of 2 with fresh TSB medium.

To separate cellular and secreted proteins, 1.6 mL of culture samples were centrifuged for 10 min at 15,800 g in an Eppendorf centrifuge at 4°C. Proteins in the resulting culture supernatant fraction were collected by precipitation with 12.5% Trichloroacetic Acid (TCA; final concentration) for 1 h on ice and subsequent centrifugation for 20 min at top speed in an Eppendorf centrifuge (4°C). After centrifugation, the supernatant was discarded, any residual TCA in the pellet was removed by washing with 1 mL of cold acetone (−20°C), and the acetone used for washing the pellet was discarded. The precipitated proteins were then dried in an Eppendorf Concentrator plus for 5 min. In the meantime, a lithium dodecyl sulfate (LDS) master mix of NuPAGE sample reducing agent 10× (Life Technologies), NuPAGE LDS sample buffer 4× (Life Technologies) and MiliQ water was prepared with a total volume of 1 mL. Of this mixture, 150 µL was added to both the bacterial cell pellet and the TCA-precipitated proteins from the culture supernatant fraction. The resuspended bacteria were then disrupted by adding a scoop of glass beads (Biospec Products, Bartlesville, USA) and bead-beating in a Precellys 24 tissue homogenizer (6500 rpm, 3 × 30s with 30 s breaks; Bertin Technologies, France). Lastly, proteins thus extracted from the bacterial cells and proteins collected from the culture supernatant by TCA precipitation were denatured at 95°C for 10 min using a Thermostat Plus (Eppendorf, Germany).

Proteins were separated in NuPAGE Bis-Tris Midi gels (NOVEX, Life Technologies) using 1× MES buffer (NuPAGE, Life Technologies) and a constant voltage (160 V) for 1 h. For each sample derived from the bacterial cells or the culture supernatant, a total volume of 10 µL was loaded per gel lane. For reference, a Precision Plus Protein All Blue Standards 10–250 kDa (BioRad) sample was separated on the same gel. Upon electrophoresis, the gels were either stained with Coomassie or used for Western blotting. For Western blotting, proteins separated by LDS-PAGE were transferred to nitrocellulose membranes (Protein nitrocellulose transfer paper, Whatman, Germany) by semi-dry blotting [42]. Particular proteins were then visualized by immunodetection with specific primary antibodies. To reduce the background signals caused by *S. aureus* SpA, the membranes were incubated for 1 h with a mixture of 20 µL Human Nanogam (50 mg/mL, Human IgG, Sanquin) and 0.1 g Bovine Serum Albumin in 10 mL 1× Phosphate-buffered saline (PBS) containing 0.1% Tween-20 (PBST). After washing, the membranes were incubated with primary antibodies for 2 h, and binding of the primary antibodies to the membranes was visualized with secondary goat anti-human or goat-anti rabbit antibodies labeled with IRDye 800CW (LI-COR Biosciences) for 1 h with shaking. Images of the blots were recorded using an Amersham Typhoon Biomolecular Imager. The human monoclonal antibody 1D9, which binds to the conserved 62-residue N-terminal domain of IsaA was used at a 1:5000 dilution to detect the IsaA protein and its degradation products [42,43].

### Sample preparation for proteome analyses

For proteome analyses, three biological replicates of *S. aureus* USA300 WT or mutant bacteria, respectively, were grown overnight at 37°C in TSB with vigorous shaking (250 rpm). TSB was chosen to culture the bacteria in order to obtain sufficient protein for our different surfacome and secretome analyses. The OD_600_ of each culture was adjusted to 2 to facilitate further sample processing. Next, 0.15 g of bacterial cells (wet weight) from each culture were collected by centrifugation in pre-weighted Falcon tubes (10,000 g, 5 min) at 4°C. The respective culture supernatant was transferred to another 50 mL Falcon tube and, to analyze the extracellular proteome, proteins were collected by TCA precipitation as described above. To analyze the bacterial cell surface-associated proteins, these proteins were enriched as previously described [44]. Briefly, the collected bacterial pellets were resuspended in 1 mL PBS (pH 8.0) with 1 mm phenylmethylsulfonyl fluoride, and the resuspended bacteria were transferred to 2 mL reaction tubes. Next, 100 µL of the membrane-impermeable crosslinker Sulfo-NHS-SS-Biotin (1 mg/sample; Thermo Fisher Scientific) was added to the suspension followed by 1 h gentle shaking on ice. After this, the bacteria were centrifuged (20,000 g, 1 min, 4°C) and the supernatant was discarded. The biotinylation reaction was stopped by washing 3 times with 1 mL PBS/500 mm Glycin. After each washing step, the bacteria were collected by centrifugation (20,000 g, 1 min, 4°C). Lastly, the bacteria were resuspended in 400 µL PBS (pH 8.0)/5% iodoacetamide (IAA) (W/V) and stored at −70°C until further processing.

Prior to disruption of the bacterial cells by bead-beating, 100 µL PBS (pH 8), 3-[(3-Cholamidopropyl) dimethylammonio]-1-propanesulfonate (CHAPS) (20%), amido-sulfobetaine-14 (ASB-14) (20%) was added to each sample. Upon bead-beating with glass beads in a Precellys 24 homogenizer (6500 rpm, 3 × 30s) the samples were incubated for 30 min on ice. Next, the cells were centrifuged (15,800 g, 15 min) and the supernatant was transferred to a new reaction tube. In the meantime, 150 µL NeutrAvidin-Agarose-Beads were equilibrated by washing them two times with 300 μL PBS/nonidet *p*-40 (octyl phenoxylpolyethoxylethanol) (NP-40). The beads were then added to the sample with biotinylated surface proteins and incubation was continued for 1 h on ice with gentle shaking. After this, the sample was centrifuged (80 g, 1 min, 4°C) and the supernatant was discarded. To remove unbound unlabeled proteins, the beads with the bound biotinylated proteins were washed four times with 1 mL PBS/NP-40 (1%)/CHAPS (6%), and vortexed for about 10 s to properly mix the beads and the washing solution. After each step, the beads were centrifuged at 80 g and 4°C for 1 min and the supernatant was discarded. The beads with the bound biotinylated proteins were washed another 2 times with 1 mL PBS/NP-40 (1%)/SDS (2%), which involved vortexing 10 s and centrifugation at 80 g and RT for 1 min. Next, 1 mL 10% mercaptoethanol elution solution was added to the sample followed by gentle shaking for 1 h at RT. Upon centrifugation at 80 g for 1 min, the supernatant was transferred to a 15 mL reaction tube with 0.925 g IAA in 3 mL distilled water. Another 1 mL elution solution was added to the beads, the sample was centrifuged (80 g, 1 min, RT) and the supernatant was also transferred to the above IAA solution. The tubes were then incubated in the dark for 20 min with gentle shaking and, subsequently, the samples were precipitated in 20 mL precooled acetone (80%, −20°C) overnight. The precipitated proteins were then collected by centrifugation (10,000 g, 30 min, 4°C), and the protein pellets were washed with 1 mL ethanol (98%, 4°C) and transferred to a 1.5 mL reaction tube. The samples were centrifuged (15,800 g, 5 min, 4°C) once more and the pellets were dried in a vacuum concentrator for 3–5 min. Then the proteins were dissolved in 15 µL urea (6 M)/thiourea (2 M) and heated for 2 min to 80°C. In the meantime, a master mix of NuPAGE sample reducing agent 10× (1:10, Life Technologies), NuPAGE LDS sample buffer 4× (1:4, Life Technologies) and MiliQ water was prepared with a total volume of 1 mL. Of this mixture, 10 µL was added to each sample. The collected surface and extracellular proteins were separated by SDS-PAGE and stained with Coomassie. Protein-containing gel lanes were excised in one piece, subjected to trypsin digestion as described previously [45], and subsequently purified with Pierce C18 tips according to the manufacturer.

### Mass spectrometry

For liquid chromatography tandem mass spectrometry (LC-MS/MS) measurements, purified protein digests were separated by reversed phase column chromatography using an EASY nLC 1200 (Thermo Fisher Scientific) with self-packed columns (OD 360 μm, ID 100 μm, length 20 cm) filled with 3 µm diameter C18 particles (Dr. Maisch, Ammerbuch-Entringen, Germany) at a constant temperature of 45°C. Following loading and desalting in 0.1% acetic acid in water, the peptides were separated by applying a binary non-linear gradient from 1–99% acetonitrile in 0.1% acetic acid over 166 min. The LC was coupled online to a LTQ Orbitrap Elite mass spectrometer (Thermo Fisher, Bremen, Germany) with a spray voltage of 2.6 kV. After a survey scan in the Orbitrap (*r* = 60,000), MS/MS data were recorded for the 20 most intense precursor ions in the linear ion trap. Singly charged ions were not considered for MS/MS analysis. The lock mass option was enabled throughout all analyses. The resulting MS data were searched against a database of *S. aureus* USA300 obtained from UniProt (UP00000193, downloaded on 13 December 2023, 2607 entries) using MaxQuant (version 2.3.1.0) [46]. MaxQuant included common laboratory contaminants and reversed sequences, and the following search parameters were used: Trypsin/P specific digestion with up to two missed cleavages, methionine oxidation and N-terminal acetylation as variable modification, match between runs with default parameters enabled. The false discovery rates (FDRs) of proteins and peptide spectrum match (PSM) levels were set to 0.01. For protein identification two identified unique peptides were required. MaxLFQ values were calculated with MaxQuant using default settings as proxy for protein abundance [47]. A principal component analysis (PCA) of the MS data was performed based on the LFQ intensities using the Scikit-Learn library for Python 3.9. The PSORTb algorithm [48] was used to predict the subcellular localization of identified proteins. “TheSEED” was used to annotate virulence genes. The AureoWiki database (https://aureowiki.med.uni-greifswald.de/) was used for functional gene categories and regulon annotation, and the WeightedTreemaps package for R was used to create Voronoi treemaps.

### Internalization experiments

A confluent layer of 16HBE14o- cells was used for bacterial internalization experiments as previously described [49]. Three days prior infection, 24-well plates were seeded with 1 × 10^5^ cells per well. On the day of the infection experiment, overnight-cultured bacteria in RPMI were diluted with fresh RPMI medium to an OD_600_ of 2. Next, the 16HBE14o- epithelial cell number in one well was counted as described above and, based on the cell count, bacteria were added to achieve a MOI of 25. For this purpose, the bacteria were diluted in eMEM and, subsequently, the eMEM with the bacteria was used to replace the culture medium of the 16HBE14o- cells. Culturing of the infected cells was continued for 1 h at 37°C with 5% CO_2_. To assess the number of internalized bacteria, the medium containing the non-internalized bacteria was removed, the cells were washed once with PBS, and fresh eMEM medium containing 25 µg/mL lysostaphin (AMBI Products, New York) was added to the cells. The lysostaphin was added to the medium in order to eliminate bacteria adhering to the surface of the 16HBE14o- cells, and to prevent reinfection of 16HBE14o- cells by initially internalized bacteria that had lysed their host cell. After incubation at 37°C for 30 min, the medium with lysostaphin was removed, PBS was used to wash the cells once and, lastly, the cells were lysed by incubation with Sodium dodecyl sulfate (SDS) for 5 min. The number of internalized bacteria was determined by serial dilution of the lysed 16HBE14o- cells in PBS and subsequent plating on Tryptic Soy agar (TSA) in triplicate for Colony Forming Unit (CFU)-counting. The internalization experiments were repeated three times.

### Statistical analyses

Statistical analyses were performed using Prism version 10, R version 4.3.1 and Python version 3.9. R studio was used for the generation of heatmaps and Voronoi treemaps. Heatmaps were plotted using the R package pheatmaps based on z-score-normalized LFQ intensities. Absent proteins were imputed with a value of − 50 to avoid missing values, allowing the classification of those proteins quantified in only one condition. On the other hand, Voronoi treemaps were generated through the WeightedTreemaps library for R, based on the log2 fold change of the LFQ intensities between the compared conditions. Cell sizes were calculated equally for all quantified proteins and grouped based on the functional categories from Aurowiki. The statistical differences in cytotoxicity and internalization rate in human lung epithelial cells between *S. aureus* USA300 WT versus individual protease mutant strains were assessed by unpaired t-tests.

## Results and discussion

### Mutations in *aur, scpA, sspA* or *sspB* cause increased cytotoxicity towards human lung epithelial cells

**Figure 2.**
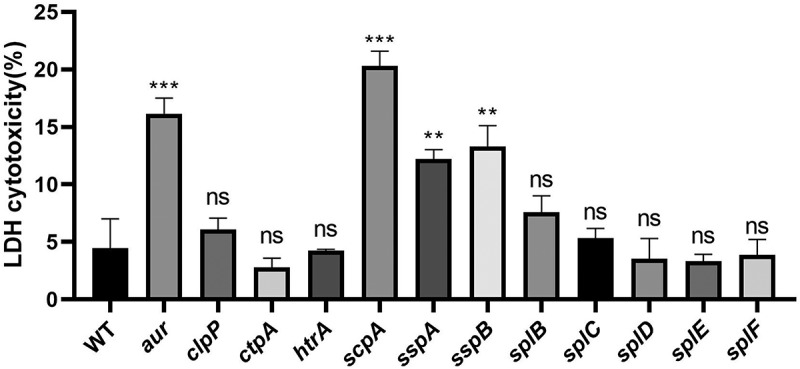
Cytotoxicity of *S. aureus* USA300 WT and single protease mutant derivatives towards human lung epithelial cells. Human lung epithelial cells were incubated with USA300 WT bacteria or single protease mutant bacteria with transposon insertions in the *aur, clpP, ctpA, htrA, scpA, sspA, sspB, splB, splC, splD, splE* or *splF* genes for 1 h at a MOI of 25. Next, the lung epithelial cell lysis was evaluated by measuring the release of LDH. The bar plots show the mean percentages from three individual experiments and the error bars show the standard deviation. The statistical significance of differences in LDH release provoked by individual mutant strains versus the WT strain was assessed by two-tailed t-tests. A P-value <0.05 was considered significant. Statistically significant differences are marked (**, *p* < 0.01; ***, *p* < 0.005; ns, not significant).

To assess the cytotoxicity of the *S. aureus* USA300 WT and 12 different single protease mutant strains toward human lung epithelial cells, the release of LDH by the epithelial cells into the cell culture medium was quantified after a 1 h bacterial challenge at a MOI of 25. Here it has to be noted that each of the 12 mutants that were available in the Nebraska transposon mutant library contained a transposon insertion in the sequences coding for the active site domain of the respective protease (Figure 1 [34]). As shown in Figure 2, the *aur*, *scpA*, *sspA* and *sspB* mutants elicited a significantly increased release of LDH compared to the WT strain, with the highest levels of LDH release being observed for the *scpA* mutant (Figure 2). In contrast, the LDH release elicited by the *clpP*, *ctpA*, *htrA*, *splB*, *splC*, *splD*, *splE* and *splF* mutants did not significantly differ from the LDH release elicited by the WT strain. Together these observations demonstrated a significantly increased cytotoxicity of the *aur*, *scpA*, *sspA* and *sspB* mutant bacteria toward human lung epithelial cells. Our subsequent analyses were therefore focused on the four proteases encoded by these genes.

### *aur, scpA, sspA* and *sspB* are involved in the degradation of IsaA during the stationary growth phase

**Figure 3.**
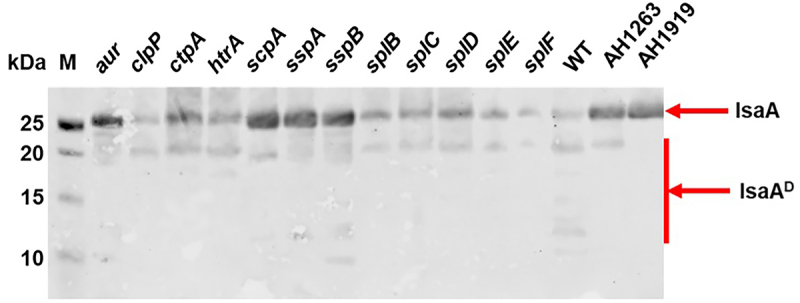
Western blotting analysis of IsaA degradation in *S. aureus* USA300 WT and single or multiple protease mutant derivatives. *S. aureus* USA300 WT, single protease mutant bacteria with transposon insertions in the *aur, clpP, ctpA, htrA, scpA, sspA, sspB, splB, splC, splD, splE* or *splF* genes, or a multiple deletion mutant lacking *aur sspA sspB* and *scpA* (AH1919) were grown overnight in TSB medium, and the presence of IsaA in the respective culture supernatant samples was analyzed by Western blotting using the IsaA-specific monoclonal antibody 1D9. The respective USA300 WT strain (WT) and the protease-proficient AH1263 were included in the analysis for control. Bands corresponding to the mature IsaA protein (marked as IsaA) and the position of IsaA degradation products are indicated. The positions of precision Plus protein molecular weight markers (in kDa), mature IsaA (marked as IsaA), and IsaA degradation products (IsaA^D^) are indicated.

To determine whether the increased cytotoxicity of the *aur*, *scpA*, *sspA* and *sspB* mutants was associated with increased proteolytic activity, we used the *S. aureus* IsaA protein as a marker, because previous research had shown that this protein is highly susceptible to proteolysis [23,50,51]. For this purpose, the 12 single protease mutant strains and the WT were grown overnight and, subsequently, culture supernatant fractions of each strain were analyzed by Western blotting with the monoclonal antibody 1D9 that specifically binds to a conserved N-terminal domain of IsaA [42,43]. Indeed, the results showed increased levels of the mature IsaA protein in the culture supernatants of the *aur*, *scpA*, *sspA* and *sspB* mutants compared to the supernatant of the WT strain (Figure 3). Concomitantly, the relative amounts of dominant IsaA degradation products were reduced in the culture supernatants of the *aur*, *scpA*, *sspA* and *sspB* mutants. In contrast, no such increase in the levels of mature IsaA, nor a reduction in the levels of IsaA degradation products, could be observed for the other single protease mutant strains that also did not show increased cytotoxicity (Figure 2 and 3). Interestingly, compared to the respective protease-proficient parental strain AH1263, IsaA degradation was virtually absent in the quadruple mutant strain AH1919 from which the *aur*, *scpA*, *sspA* and *sspB* had been deleted (Figure 3). On this basis, we conclude that absence of the proteolytic activity of the Aur, ScpA, SspA and SspB proteases results, directly or indirectly, in the increased cytotoxicity of the *aur*, *scpA*, *sspA* and *sspB* mutant strains.

### Surfacome and exoproteome analysis of *S. aureus* USA300 WT and the *aur, scpA, sspA* or *sspB* mutant strains

To investigate how the *aur*, *scpA, sspA* or *sspB* mutations impact on the surfacome and secretome of *S. aureus* USA300, we performed proteomics analyses. To this end, the bacteria were grown overnight in TSB and, on the next day, cells were separated from the growth medium by centrifugation. Proteins in the resulting culture supernatant fractions were collected by TCA precipitation to chart the secretomes of the different strains. In contrast, the surfacome proteins of the collected cells were biotinylated with the membrane-impermeable crosslinker Sulfo-NHS-SS-Biotin and subsequently purified with NeutrAvidin-Agarose-Beads. The secretome and surfacome fractions thus obtained were then analyzed by LC-MS/MS. The resulting data are summarized in Supplemental Table S1.

**Figure 4.**
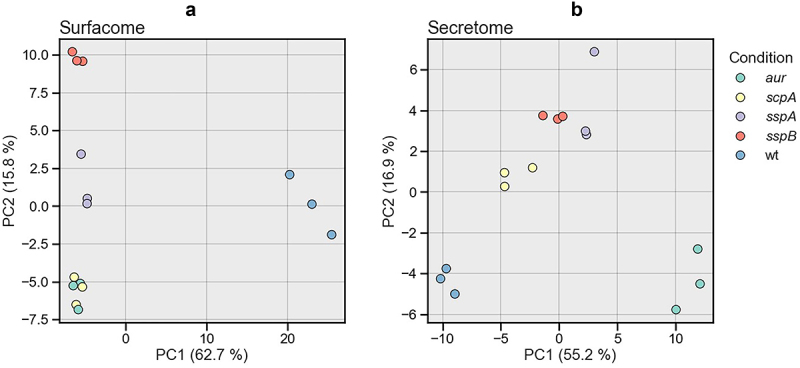
Principal component analyses (PCAs) of identified surface-located (a) and extracellular (b) proteins of *S. aureus* USA300 WT and *aur*, *scpA*, *sspA*, or *sspB* single mutant strains. The PCA was performed based on the LFQ intensities using the Scikit-Learn library for Python 3.9. Three biological replicates of each strain were analyzed and the respective data points are marked with the same color-code in panels A and B.

To evaluate the quality of the MS data and to visualize possible variations between the *aur*, *scpA*, *sspA*, *sspB* mutants and the WT, principal component analyses (PCA) were performed. The PCA results showed that the three biological replicates of each individual investigated strain clustered closely together, indicating high consistency of the MS data obtained for each strain (Figure 4). In contrast, the replicate data obtained for the four protease mutant strains showed a clear separation from the WT and, in most cases, from each other (Figure 4). Only the surfacome data obtained for the *aur* and *scpA* mutant bacteria clustered together, implying that the two protease mutations have very similar effects on the composition of the surfacome. Altogether, the PCA analyses revealed significant differences in the protein compositions of the surfacomes and secretomes of the four protease mutants. Notably, this is the first time that a profound effect of mutations in individual protease genes on the composition of the surface proteome is documented.

**Figure 5.**
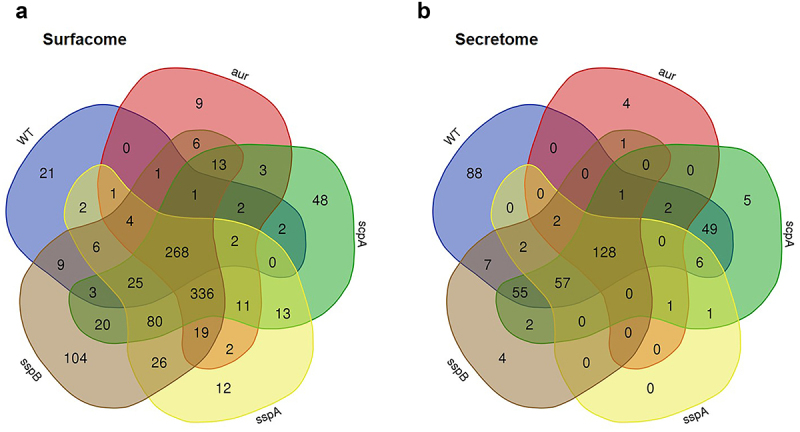
Venn diagrams illustrating the common and uniquely identified proteins in the surfacomes (a) and secretomes (b) of the *S. aureus* USA300 WT and the *aur*, *scpA*, *sspA* or *sspB* mutants. Venn diagrams were generated using the online venn tool (https://bioinformatics.Psb.ugent.be/webtools/venn/). The numbers of all proteins identified for each strain are detailed in Supplemental table S1.

In total, we identified 1057 proteins in the surfacome and 419 proteins in the secretome samples (Table S1). The numerical differences in protein identifications in the surfacomes and secretomes of the four protease mutant strains compared to the WT strain are visualized in the Venn diagrams in Figure 5. In particular, we identified 268 common surfacome proteins and 128 common secretome proteins, whereas 789 surfacome proteins (i.e. 75%) and 291 secretome proteins (~69%) were variable. Furthermore, we identified 232 shared surfacome proteins that were only detected in the four protease mutant strains, but not in the WT USA300 strain (Supplemental Figure S1). In contrast, none of the secretome proteins identified only in the four protease mutants was shared by these mutants (Supplemental Figure S1). Compared to the WT surfacome, 459 additional surfacome proteins were identified in the *sspB* mutant, 399 in the *scpA* mutant, 384 in the *sspA* mutant, and 275 in the *aur* mutant (Supplemental Table S1). Furthermore, compared to the WT secretome, 3 additional secretome proteins were identified in the *sspB* mutant, 1 in the *scpA* mutant, 1 in the *sspA* mutant, and 3 in the *aur* mutant.

Importantly, the surfacomes and secretomes of the protease mutants did not only differ in terms of uniquely identified proteins, but also in the relative abundance of shared surfacome and secretome proteins. As detailed in Supplemental Table S1, 85 surfacome proteins showed a significantly different relative abundance in one or more protease mutants compared to the WT, and the same was true for 11 secretome proteins. Overall, the largest differences compared to the WT were observed in the surfacome of the *sspB* mutant, where 604 proteins were identified uniquely or with altered abundance.

### Predicted subcellular localization of identified surfacome and secretome proteins of *S. aureus* USA300 WT and the *aur, scpA, sspA* or *sspB* mutants

**Figure 6.**
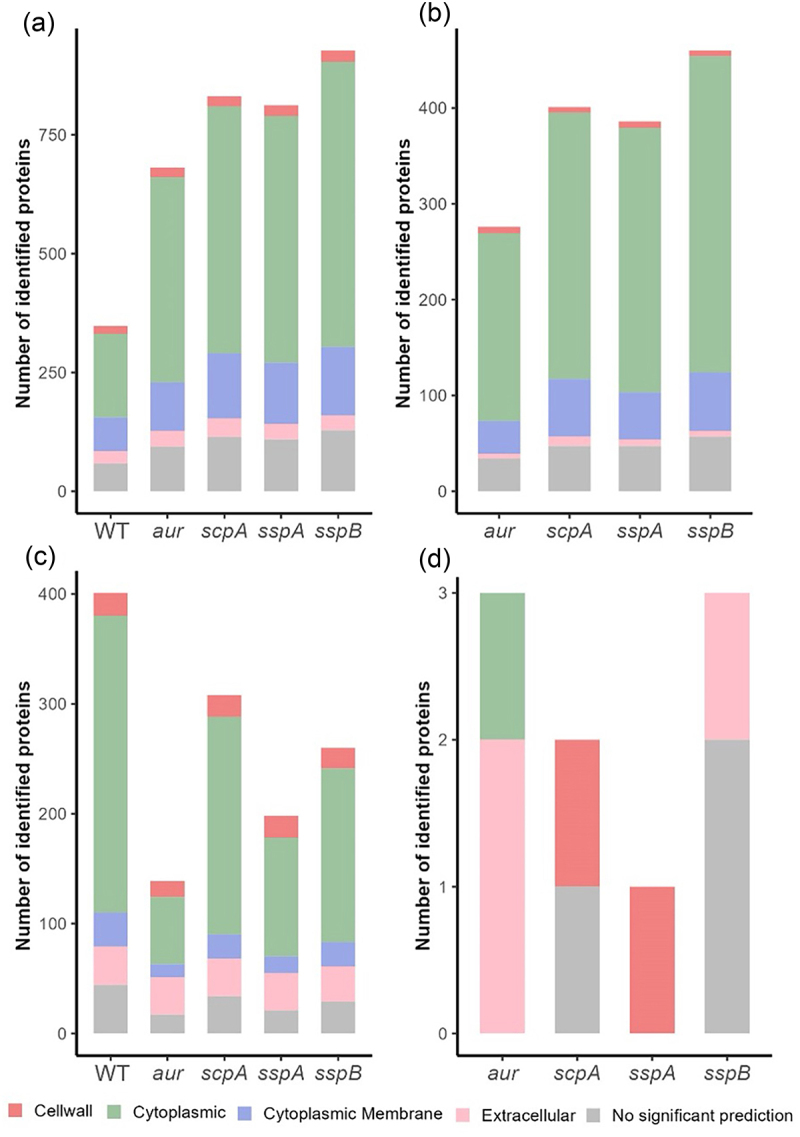
Overview of the predicted subcellular localizations of identified proteins in the *S. aureus* USA300 WT and the *aur*, *scpA*, *sspA* or *sspB* mutant strains. Using PSORTb, the subcellular localizations were predicted for (a) all identified surfacome proteins, (b) proteins identified only in the surfacome fraction of the *aur*, *scpA*, *sspA*, *sspB* mutants but not in the WT surfacome, (c) all identified secretome proteins, and (d) proteins identified only in the secretome fraction of the four protease mutants but not in the WT secretome. Panels (a–d) present the numbers of proteins with a predicted subcellular or extracellular location in color code. Please note the different ranges of the y-axes of the four panels.

To determine the possible origins of the large numbers of additional proteins that we identified for the four protease mutant strains compared to the USA300 WT strain, we predicted the subcellular localization of all proteins identified for these strains according to the PSORTb algorithm. The results are presented in Supplemental Table S1 and summarized in Figure 6. In particular, the identified proteins were categorized for having a predicted cytoplasmic, membrane, cell wall or extracellular localization. Of note, for some proteins no reliable localization prediction could be obtained with PSORTb. As evidenced in Figure 6, panels A and B, the numbers of surfacome proteins with a predicted cytoplasmic or cytoplasmic membrane localization were significantly increased in the protease mutant strains and the same was observed for proteins with a localization that could not be predicted with confidence. Interestingly, in the secretome we observed the opposite trend, namely a reduction in the numbers of identified proteins with a predicted cytoplasmic or cytoplasmic membrane localization (Figure 6(c,d)). This was particularly evident for the *aur* mutant strain. Together, these data show that the large numbers of additional proteins that we identified in the four protease mutant strains compared to the USA300 WT strain are most likely related to increased cell leakiness or lysis due to the protease mutations. Furthermore, the apparent relocation of proteins released from the cytoplasm or the membrane by leakiness or lysis of the protease mutant bacteria to the surfacome of the surviving bacteria suggests that, in the absence of Aur, ScpA, SspA or SspB, the bacterial cell surface has an increased capacity to bind released cytoplasmic proteins. As for the identified secretome proteins, the Sak protein which is known to be actively secreted [6] was identified only in the secretome of the *aur* mutant.

**Figure 7.**
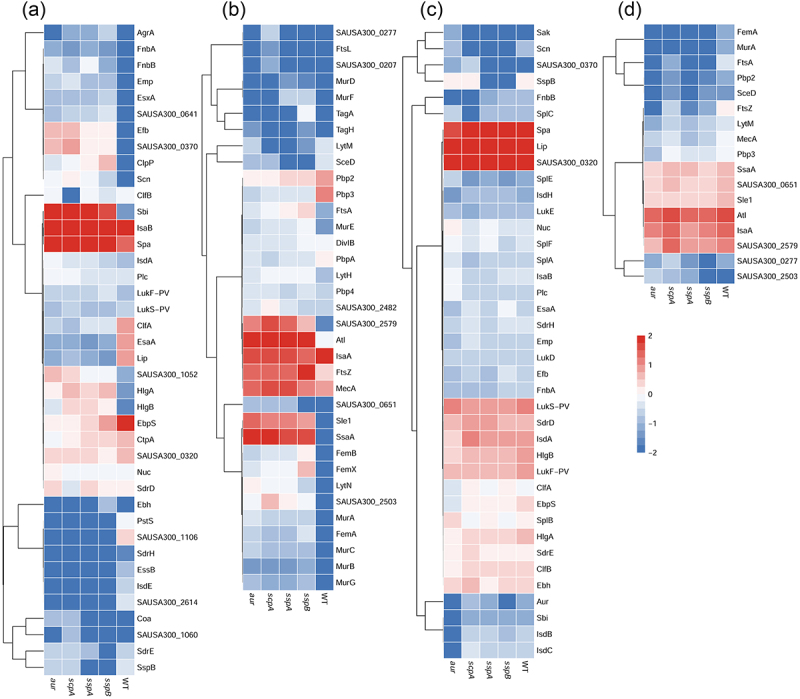
Heatmap representation of the relative abundance of the known *S. aureus* virulence factors and proteins involved in cell wall synthesis and turnover as identified in the surfacome (a-b) and secretome (c–d) of *S. aureus* USA300 WT and the *aur*, *scpA*, *sspA* or *sspB* mutant strains. A total number of 39 virulence factors (a) and 35 proteins involved in cell wall synthesis and turnover (b) was identified in the investigated surfacomes, and 38 known virulence factors (c) and 17 proteins involved in cell wall synthesis and turnover (d) were identified in the secretomes of the *S. aureus* USA300 WT and the *aur*, *scpA*, *sspA* and *sspB* mutants. The abundance of the included virulence factors in the different bacteria was determined based on Z-Score normalized LFQ intensity. Protein levels are indicated by the respective color coding. Deep blue (<−2.5 intensity) represents proteins highly reduced or absent in the investigated strains. The proteins were sorted by hierarchical clustering.

Considering the increased numbers of cytoplasmic and cytoplasmic membrane proteins released to the surfacome of the four protease mutants, we checked whether these increases could potentially be attributed to elevated levels of peptidoglycan hydrolases (PGHs). If so, increased PGH activity could lead to elevated levels of cell leakiness or lysis, and consequently, release of cytoplasmic and cytoplasmic membrane proteins into the extracellular environment as was previously observed for the Gram-positive bacterium *Bacillus subtilis* [52]. Indeed, the PGHs SsaA and Sle1, which were previously implicated in *S. aureus* cell lysis [39,53], were identified in the surfacome fractions of the four protease mutants, but not in the WT surfacome (Figure 7(b); Supplemental Table S1). In addition, the levels of the PGHs Atl and LytN, and the “cysteine, histidine-dependent amidohydrolase/peptidase” (CHAP) PGH SAUSA300_2503, which were also previously implicated in cell lysis [39,54,55], were increased in the surfacome of the protease mutant strains (Figure 7(b)). Furthermore, the PGH SAUSA300_2503 was identified in the secretome of the *aur*, *scpA* and *sspA* mutants, but not in the secretome of the WT and the *sspB* mutant strains (Figure 7(b,d)). Altogether, these observations show that, compared to the WT strain, the *aur*, *scpA*, *sspA* and *sspB* mutants have higher levels of PGHs in their surfacomes and secretomes. Accordingly, these observations indicate that the four protease mutant strains have an increased propensity for autolysis compared to the WT strain, which would explain why relatively more cytoplasmic and cytoplasmic membrane proteins were identified in the surfacome fractions of the four mutant strains compared to the WT strain. Furthermore, these observations indicate that the Aur, ScpA, SspA and SspB proteases may cooperate in the degradation of PGHs, thereby suppressing cell wall remodeling and degradation.

Notably, proteins identified only in the surfacome and secretome fractions of the four protease mutants but not in the respective fractions of the WT strain, especially PGHs like LytN and SAUSA300_2503, are likely to be substrates of the respective inactivated proteases. This implies the existence of a self-enhancing effect with respect to the accumulation of cytoplasmic and cytoplasmic membrane proteins on the bacterial cell surface and in the extracellular medium, where reduced control of PGHs by proteases leads to enhanced bacterial cell leakiness or lysis and shedding of cytoplasmic and cytoplasmic membrane proteins that, in turn, are less effectively degraded due to the absence of critical proteases.

### Function of identified surfacome and secretome proteins of S. aureus USA300 and the *aur, scpA, sspA* and *sspB* mutants

To profile the functions of the identified surfacome and secretome proteins of *S. aureus* USA300 WT and the *aur*, *scpA*, *sspA* or *sspB* mutants, overviews of the biological functions of the unique or differentially abundant proteins of the *aur*, *scpA*, *sspA* or *sspB* mutant bacteria are presented in the form of Voronoi treemaps (Supplemental Figure S2). These treemaps assign functionally related proteins to the same cluster and partition them into weighted polygons with an area proportional to the relative weight of the respective functional category based on the functional “TheSEED” annotation of *S. aureus* genes. The Voronoi treemaps show that the differentially identified surfacome proteins of the *aur*, *scpA*, *sspA* or *sspB* mutant bacteria compared to WT belong to five main functional protein categories, namely (i) protein metabolism, (ii) carbohydrates, (iii) amino acids and derivatives, (iv) cell wall and capsule, and (v) virulence, disease, and defense. Furthermore, the Voronoi treemaps highlight the remarkable finding that the identified surface proteins of the *aur*, *scpA*, *sspA* and *sspB* mutants have distinct functional signatures, although for a relatively large group of identified proteins the function has not yet been identified. Similarly, the differentially identified secretome proteins of the *aur*, *scpA*, *sspA*, or *sspB* mutant bacteria compared to the WT, fall predominantly into the same five functional protein categories.

The differential levels of surfacome and secretome proteins of the WT USA300 strain and the *aur*, *scpA*, *sspA* or *sspB* mutants that have functions in virulence and cell wall homeostasis are graphically represented in the heat maps of Figure 7. A total number of 52 known virulence factors of *S. aureus* were identified, of which 13 were identified exclusively in the surfacome (Figure 7(a)) and 12 exclusively in the secretome (Figure 7(c)). These virulence factors are involved in bacterial adhesion, cytotoxicity, antibiotic resistance, biofilm formation and immune evasion [6]. Importantly, several virulence factors involved in adhesion, like FnbA, FnbB, SAUSA300_1052, Emp and Efb, were overrepresented in the surfacome of protease mutants compared to the WT strain, and the same applied for the immune evasion factors Sbi and SpA, and the HlgA and HlgB components of the bicomponent cytolytic HlgAB toxin (Figure 7(a)). Furthermore, the staphylococcal complement inhibitor (SCIN, Scn) was detected in the surfacome of the four protease mutants, but not in the surfacome of the WT strain. Together, these observations are sufficient to explain the increased cytotoxicity of the four protease mutant strains toward human lung epithelial cells (Figure 2).

Lastly, the heatmaps in Figure 7 draw attention to a few additional observations. First, SspB was not detected in the surfacome and secretome of the *sspA* mutant, which suggests that the *sspB* gene was not expressed due to the transposon insertion in the upstream *sspA* gene (Figure 1). Second, the increased abundance of mature IsaA in the culture supernatant of the four protease mutant strains, as detected by Western blotting (Figure 2) was not mirrored by the MS data. We attribute this observation to the fact that IsaA is not fully degraded in the WT, but that particular cleavage products accumulate in the growth medium (Figure 2), which are detected by MS. Third, the elevated levels of several proteins in the surfacomes and secretomes of the *aur*, *scpA*, *sspA* and *sspB* mutants compared to the WT imply that the encoded Aur, ScpA, SspA and SspB proteases cooperate in the degradation of these proteins. This view is supported by the Western blotting results for IsaA, which showed that IsaA degradation was only undetectable in a quadruple *aur*, *scpA*, *sspA*, *sspB* protease mutant (Figure 2).

### Peptide mapping highlights differential cleavage of *S. aureus* proteins in the surfacome and secretome

For protein identification based on MS, we used the identification of two unique peptides per protein as the key criterion. However, the MS data set for many of the presently identified proteins is much richer in information due to the identification of more than two peptides per protein. This allowed us to also investigate possible differences in peptide identifications in the *aur*, *scpA*, *sspA* and *sspB* mutants compared to the WT strain (Supplemental Table S2). To visualize such differences, we mapped for all proteins identified in the surfacomes and secretomes of the investigated strains the identified peptides against the amino acid sequences of the proteins from which these peptides are derived. The complete set of the resulting data from the surfacome and the secretome samples is shown in Supplemental Figures 3 and 4, respectively. Based on these results several patterns can be distinguished that can be directly or indirectly linked to differential protease activities in the WT and the mutant strains.

**Figure 8.**
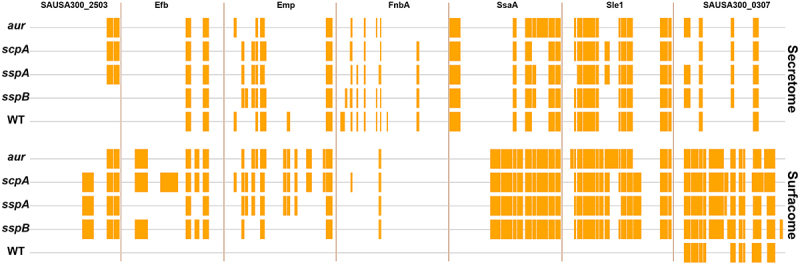
Mapping of peptides identified by MS on the amino acid sequence of representative surfacome and secretome proteins in the *S. aureus* USA300 WT strain and the *aur*, *scpA*, mutant strains. Peptides of the SAUSA300_2503, Efb, Emp, FnbA, SsaA, Sle1 and SAUSA300 proteins as identified through proteomics in the surfacome and secretome fractions of the *S. aureus* USA300 WT and the *aur*, *scpA*, *sspA* or *sspB* mutants are marked as orange blocks in the linearly depicted protein sequence.

A typical result obtained for proteins which were not detected in samples of the WT strain, but that were detected in the protease mutants, is exemplified by SAUSA300_2503 (Figure 8). For this protein, C-terminally located peptides were detected upon analysis of the surfacomes and secretomes of the *aur*, *scpA*, and *sspA* mutants. While these peptides were also detected for SAUSA300_2503 in the surfacome of the *sspB* mutant, they remained undetected upon analysis of the secretome fraction of this mutant. Additional peptides of SAUSA300_2503 were detected in the surfacome fractions of the *scpA*, *sspA* and *sspB* mutants. These data suggest that the different protein domains of SAUSA300_2503 for which peptides are detected in the mutants, but not in the WT, are cleaved by the Aur, ScpA, SspA and/or SspB proteases in the WT strain.

Furthermore, peptides of the proteins Efb, Emp, FnbA, SsaA and Sle1 were not detected upon analysis of the surfacome of the WT strain, but were detectable upon analysis of the secretome of this strain. However, the identified peptide profiles observed for these proteins displayed intriguing similarities and differences (Figure 8). For instance, essentially the same peptide profiles were observed in the secretome profiles of the Efb protein in all strains, while the maps of identified Efb peptides from the surfacome analyses showed considerable variations. The largest number of Efb peptides were identified for the *scpA* mutant, suggesting that Efb is a major target for degradation by ScpA. A different effect was observed for FnbA, where few peptides were identified upon analysis of the surfacome fractions, whereas diverse peptide profiles were identified for FnbA in the secretome fractions of the WT and the four protease mutant strains (Figure 8). This suggests that most FnbA degradation by the Aur, ScpA, SspA and SspB proteases occurs on the bacterial cell surface, and differential cleavage of FnbA by one or more other proteases occurs upon release into the culture medium. Remarkably, the broadest spectrum of FnbA peptides was detected for the secretome fraction of the WT strain, suggesting that the absence of Aur, ScpA, SspA and SspB may lead to increased FnbA cleavage by other proteases. In the case of SsaA, we observed comparable peptide profiles upon analysis of the surfacome of the four protease mutant strains, but different peptide profiles upon analysis of the secretome fraction (Figure 8). In this case, additional SsaA-specific peptides detected in the secretome fractions of the *aur*, *sspA* and *sspB* mutants are suggestive of significant contributions of Aur, SspA and SspB to SsaA cleavage upon release from the cells into the growth medium. For SAUSA300_0370 (SelX), we detected the most peptides in the surfacome fraction, suggesting that this protein is relatively well protected against proteolysis in the cell wall environment, albeit that additional peptides were detected in the surfacome fractions of the four protease mutant strains. On the other hand, much fewer SAUSA300_0370-specific peptides were detected in the secretome fraction, suggesting cleavage of this protein upon its release into the growth medium.

The peptide profiles of Emp, Sle1 and SAUSA300_0307 showed variations upon analysis of both the surfacome and secretome fractions, with considerable differences for the protease mutant strains (Figure 8). This suggests that the Aur, ScpA, SspA and SspB cleavage sites of these proteins are differentially exposed on the bacterial cell surface and in the secretome. However, it is important to note that the absence of certain peptide identifications from the WT strain and the identification of the respective peptides in one or more protease mutants is suggestive of proteolysis of particular proteins or their domains, but that there may also be other reasons why particular peptides are not identified in the MS analyses. For instance, for the proteins in the surfacome fraction it is possible that some variations in the respective peptide identifications originated from differential crosslinking with the Sulfo-NHS-SS-Biotin due to a possibly differential accessibility of crosslinkable sites of the different identified proteins in the cell wall of *S. aureus*. Furthermore, as mentioned above, it is possible that particular protease mutations may affect particular PGHs, which may result in altered release of proteins from the cell wall into the culture supernatant. Alternatively, an altered peptidoglycan structure due to altered PGH abundance may lead to retention of certain proteins in the cell wall. For instance, the covalently cell wall-attached ClfB protein was detected in the surfacome and secretome fractions of all investigated strains, except for the surfacome fraction of the *scpA* mutant. This suggests that alterations in the cell wall or increased cell wall turnover in the absence of ScpA led to the release of ClfB into the culture supernatant. Indeed, the CHAP-domain-containing PGH SAUSA300_0277 (EssH) was detected only in the cell wall of the *scpA* mutant, which might explain the release of ClfB by this strain (Figure 7). A similar mechanism was previously proposed for the PGH-mediated release of Spa from the *S. aureus* cell wall [56]. Lastly, it is conceivable that the mutation of one particular protease elicits elevated levels of another protease in the surfacome or secretome, albeit that we did not detect this effect. Thus, an alternative explanation for the absence of the cell-associated ClfB protein in the *scpA* mutant would be that this is due to an enhanced activity of an as yet unidentified alternative protease. This would not be the quality control protease ClpP as the ClpP levels were enhanced in the surfacome fractions of all four protease mutant strains. The latter observation could imply that these mutants are to some extent experiencing protein (un)folding stress, as ClpP is known to be responsible for the degradation of unfolded or misfolded proteins [57,58].

### Mutations in *aur, scpA, sspA* or *sspB* result in increased invasion into human lung epithelial cells

**Figure 9.**
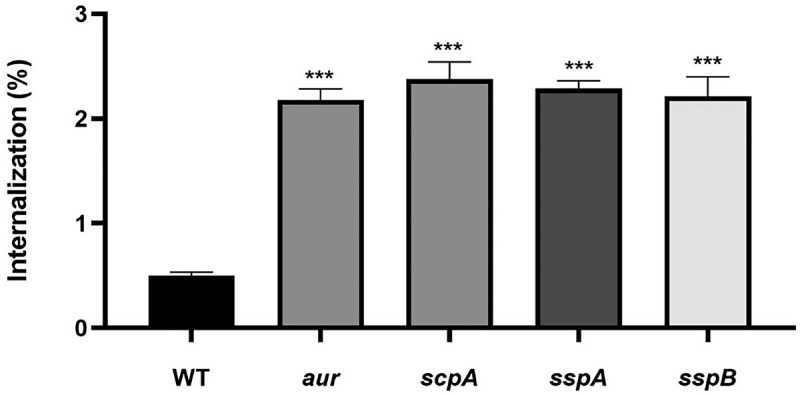
Invasive behavior of *S. aureus* USA300 WT and the *aur*, *scpA*, *sspA* or *sspB* mutant strains towards human lung epithelial cells. The cells were infected with ~ 38,400 CFUs of the different bacterial strains and the numbers of internalized WT or single protease mutant bacteria were determined after 1 h of infection and 30 min incubation with lysostaphin. To this end, the cells were lysed with 0.05% SDS and the released bacteria were collected and counted by plating on TSA plates. The internalization of the WT and mutant bacteria is presented as the proportion of the total bacterial CFU count used for the infection. The actual counted CFU numbers are presented as Supplemental table S3. The bar plot shows the mean values from three individual experiments and the error bars show the respective standard deviation. Statistical significance of the difference in epithelial cell internalization by individual protease mutant strains versus the WT strain was assessed by two-tailed t-tests. A P-value <0.05 was considered significant (***, *p* < 0.005).

Since we showed by our surfacome and secretome analyses that the *aur*, *scpA*, *sspA* and *sspB* single protease mutants of *S. aureus* USA300 displayed elevated levels of virulence factors for host cell adhesion and invasion, and possibly intracellular survival, infection experiments with 16HBE14o- human lung epithelial cells were performed to measure bacterial internalization. To this end, the 16HBE14o- cells were incubated with the WT *S. aureus* USA300 strain or the different protease mutants for 1 h at a MOI of 25, after which the bacterial internalization was measured by bacterial CFU counting. As anticipated based on the proteome analyses, all four protease mutant strains showed significantly (i.e. more than 4-fold) higher levels of internalization than the WT strain (Figure 9). These observations show that the absence of the Aur, ScpA, SspA, or SspB proteases does not only lead to increased cytotoxicity as shown in Figure 2, but also enhances the invasion into human lung epithelial cells.

## Conclusion

Proteins that are located on the surface of bacterial pathogens and proteins secreted by these bacteria into their extracellular environment play vital roles in the pathogen-host interactions that lead from host contamination to colonization and infection. For instance, surface-located proteins serve as virulence factors that contribute to tissue adhesion, immune evasion and host invasion, whereas secreted proteins have roles in the subversion of host barriers and immune cells [6,19,28]. Proteases represent a specific class of bacterial virulence factors that can serve different defensive and offensive tasks. On the one hand, they may serve to degrade antimicrobial proteins and peptides from the host, including complement C3 and immunoglobulins, and they may also cause severe damage to host cells and tissues [21,31,37,59–62]. On the other hand, bacterial proteases have important tasks in protein quality control, the regulation of cellular processes, and the nutrient acquisition from protein-rich environments [63–65]. In the present study, we investigated to what extent 12 different proteases contribute to the virulence of *S. aureus* USA300 by making use of strains from the Nebraska transposon library [34]. The results show that four of these mutants with transposon insertions in the *aur*, *scpA*, *sspA* or *sspB* genes display increased cytotoxicity toward human lung epithelial cells and an enhanced invasive behavior. By following a proteomics approach, we correlate these increases in cytotoxicity and invasion to altered profiles of virulence factors in the surfacomes and secretomes of these four mutant strains. This implies that the Aur, ScpA, SspA and SspB proteases actually suppress the virulence of *S. aureus* USA300 and that the effects of their individual elimination result in at least partially overlapping phenotypes. These observations are consistent with the results of a previous study by Gimza *et al*., who showed that an *aur scpA* double mutant presented a hypervirulent phenotype in a murine sepsis model [19]. Furthermore, our present results align well with previous observations by Kolar et al. [31], who reported contrasting roles for *S. aureus* proteases in a murine infection model with respect to morbidity and mortality. In particular the absence of extracellular proteases was shown to increase the levels of several secreted and surface-associated virulence factors, which was associated with increased mortality [31]. Conversely, it was recently shown that mutation of the SarA gene regulator of *S. aureus* led to attenuation in a murine osteomyelitis model due to increased extracellular protease production [66,67].

Based on our present data, it is difficult to determine whether the suppression of virulence by Aur, ScpA, SspA and SspB in *S. aureus* represents (i) an evolved strategy that avoids hypervirulent phenotypes, which could reduce the pathogen’s fitness by premature killing of the host, or (ii) whether production of the four proteases is a beneficial evolutionary “trade-off” that helps the bacteria to maintain a balance between virulence, nutrient acquisition, protein quality control and the control of PGHs at the bacterial cell surface. The latter activity of the four proteases is underscored by the observed cleavage of the lytic transglycosylase IsaA, but also by the observed effects on the levels of other PGHs, such as Atl, LytN, SAUSA300_0277, SAUSA300_2503, Sle1 and SsaA. However, our results show that not only PGHs are controlled by Aur, ScpA, SspA and SspB, but they also show for the first time that these four proteases have a profound impact on the surfacome as a whole. A particularly remarkable finding is that, in absence of either one of these four proteases, many proteins are apparently relocated from the secretome to the surfacome. In particular, 232 common surface-associated proteins were identified in the 4 protease mutants, but not in the WT strain, suggesting that they are absent from the WT surfacome due to proteolytic cleavage and subsequent degradation or release from the cell wall into the extracellular environment. Furthermore, the observed accumulation of proteins in the cell wall of the protease mutant strains may relate to changes in their cell wall composition, both at the levels of cell wall polymers and the presence or absence of particular proteins. Lastly, it is relevant to note that the Aur, ScpA, SspA and SspB proteases seem to cooperate in the cleavage and/or degradation of many of the identified virulence factors and proteins contributing to cell wall biogenesis and turnover. This probably relates to the fact that Aur is involved in the activation of SspA and that SspA is, in turn, involved in the activation of SspB [12].

While our present study provides new insights into the individual roles of the Aur, ScpA, SspA and SspB proteases in shaping the surfacome and secretome of *S. aureus*, which are relevant for our understanding of staphylococcal virulence, some limitations need to be acknowledged. Firstly, our proteomic analyses were conducted on bacteria and their secreted proteins upon *in vitro* culture in TSB medium. This culture condition was chosen for practical reasons, but it does not fully replicate the complex environment encountered during host infection [26,28,40]. Future studies should therefore include proteomic profiling of *S. aureus* protease mutants under physiologically more relevant conditions, such as cell culture media and other media that mimic the different extra- and intracellular human host niches occupied or invaded by *S. aureus* [68]. Secondly, since transposon insertions lead to gene truncations, it is conceivable that expression of some of the resulting truncated proteins could contribute to some of the observed phenotypes. In addition, the transposon insertions may impact the expression of downstream genes, as suggested by the possibly polar effect of transposon insertion in the *sspA* gene on expression of the downstream *sspB* gene. While such indirect or polar effects may also occur in nature by spontaneous mutation or transposon insertion, they can be ruled out in future studies by constructing deletion mutants that lack the entire genes from start to stop codon. Additionally, whole-genome sequencing could then be applied to rule out the possibility of emerging suppressor mutations. Lastly, as was previously shown, the proteolytic enzymes of *S. aureus* participate in a complex network of processing interactions [12,15,69]. This makes it difficult to distinguish direct from indirect effects of individual protease mutations, irrespective of how these mutations are created. Unfortunately, it will be very challenging to reproduce *in vitro* the complexity of such interactions and their impacts on the composition of the *S. aureus* surfacome and secretome with individual purified components. Yet, this will be an important objective for future studies.

Altogether, based on our present observations, we conclude that the secreted proteases Aur, ScpA, SspA and SspB have major roles in shaping the surfacome and secretome of *S. aureus*. As a consequence, they control the virulence of this major opportunistic human pathogen. Their absence leads to hypervirulent bacteria that exhibit increased cytotoxicity and host cell invasion. In turn this means, that novel antimicrobial agents or vaccines should preferably not target Aur, ScpA, SspA and/or SspB.

## Data Availability

The MS proteomics data discussed in this publication have been deposited to the ProteomeXchange Consortium via the PRIDE partner repository at http://www.ebi.ac.uk/pride [70] with the dataset accession number P×D056653 (https://www.ebi.ac.uk/pride/archive/projects/PXD056653).
